# Top-ranked expressed gene transcripts of human protein-coding genes investigated with GTEx dataset

**DOI:** 10.1038/s41598-020-73081-5

**Published:** 2020-10-01

**Authors:** Kuo-Feng Tung, Chao-Yu Pan, Chao-Hsin Chen, Wen-chang Lin

**Affiliations:** 1 Institute of Biomedical Sciences, Academia Sinica, Taipei, 115 Taiwan, ROC; 2 Institute of Biomedical Informatics, National Yang-Ming University, Taipei, Taiwan, ROC

**Keywords:** Genetic databases, Computational biology and bioinformatics, Sequence annotation

## Abstract

With considerable accumulation of RNA-Seq transcriptome data, we have extended our understanding about protein-coding gene transcript compositions. However, alternatively compounded patterns of human protein-coding gene transcripts would complicate gene expression data processing and interpretation. It is essential to exhaustively interrogate complex mRNA isoforms of protein-coding genes with an unified data resource. In order to investigate representative mRNA transcript isoforms to be utilized as transcriptome analysis references, we utilized GTEx data to establish a top-ranked transcript isoform expression data resource for human protein-coding genes. Distinctive tissue specific expression profiles and modulations could be observed for individual top-ranked transcripts of protein-coding genes. Protein-coding transcripts or genes do occupy much higher expression fraction in transcriptome data. In addition, top-ranked transcripts are the dominantly expressed ones in various normal tissues. Intriguingly, some of the top-ranked transcripts are noncoding splicing isoforms, which imply diverse gene regulation mechanisms. Comprehensive investigation on the tissue expression patterns of top-ranked transcript isoforms is crucial. Thus, we established a web tool to examine top-ranked transcript isoforms in various human normal tissue types, which provides concise transcript information and easy-to-use graphical user interfaces. Investigation of top-ranked transcript isoforms would contribute understanding on the functional significance of distinctive alternatively spliced transcript isoforms.

## Introduction

Significant advances in genome sequencing projects have considerably transformed the extent of biomedical and bioinformatic researches^1^. Complete genome sequences would promote the comprehension on the whole genome composition; gene loci structures and their regulation. Intriguingly, sequence determination of complex genomes, which was once considered unthinkable, is now the easiest component of any given genome project due to the incredible advancements in next-generation sequencing (NGS) platforms^2^. Among the sequenced organisms, the human genome project was declared complete in 2003 as a celebration of the 50th anniversary of the DNA double helix structure unveiling^3^. While new advancement on the protein-coding gene prediction has been obtained with deep-learning based machine learning approaches^4,5^, however, comprehensive decipher of human protein-coding gene repertoire is remained a challenging task and needs manual curation from genome biologists^6^. One major difficulty confronted is complex alternatively spliced transcript isoforms in human protein-coding genes. Furthermore, annotation discrepancy can be found among different bioinformatic and genomic databases. Therefore, information regarding reference annotated mRNA transcripts of respective human protein-coding genes would be beneficial for biomedical researches and pathological sequence variation analyses.

With the applications of NGS platform in transcriptome studies and accumulation of NGS reads, the breadth and diversity of human transcriptome are found to be dynamic and more extensive than previously recognized^7,8^. Numerous large-scale RNA-Seq transcriptome studies, such as the Genotype-Tissue Expression (GTEx)^9^, Cancer Cell Line Encyclopedia (CCLE)^10^, and The Cancer Genome Atlas (TCGA)^11^, have accumulated massive quantities of human gene expression information in tissues and pathological conditions. These studies provided us more information on the spliced transcript isoforms of protein-coding genes as well as more understanding on their expression profiles and translated protein products in human tissues and diseases. On the other hands, newly developed RNA-Seq methods, such as CaptureSeq, demonstrated to be effective in greatly enrich the sequencing depth on given transcriptome studies^12,13^. Even with the increasing sequencing depth of transcriptome studies, elucidating the full extent of transcriptome depth and tissue variation is still a challenging effort ahead. There are even more alternatively spliced isoforms discovered in human protein-coding genes and noncoding genes. Therefore, it is essential to establish user-friendly data resources for interrogation on the expressed alternatively spliced transcript isoforms of human protein-coding genes. Researchers would be able to have better realization on the expression of each alternatively spliced transcript isoforms of human protein-coding genes and choose appropriate reference transcripts for subsequent NGS data analysis pipelines. Previously, our laboratory has successfully employed the RefSeq gene collection for miRNA and gene discovery studies and demonstrated the importance of reference gene sequence information in evolution genomic studies and NGS expression analysis^14–16^. We have learned the significance and usefulness of profound reference gene sequences (such as RefSeq^17^).

Therefore, a new joint MANE project (*M*atched *A*nnotations from *N*CBI and *E*BI) was established recently by two major bioinformatic centers (NCBI and EBI) to resolve the inconsistent human protein-coding gene annotations (https://www.ncbi.nlm.nih.gov/refseq/MANE/). Previously, the RefSeq dataset from the NCBI^17^ and the Ensembl project from the EBI^18^ have been the gold standard datasets for high-quality human genome, gene, and transcript reference data and have been used by scientists worldwide. The MANE project aims to thoroughly inspect the RefSeq and Ensembl/GENCODE human gene collections and standardize new subsets of transcripts per gene to create a new reference standard because the current foremost problem is the complex patterns of large numbers of alternatively spliced mRNA isoforms. The MANE project emphasizes the importance of the creation of more unified and single representative gene transcript annotation for human protein-coding genes. Therefore, consolidating and identifying a putative single representative reference transcript for each protein-coding gene is a massive effort. While we believe that this MANE-select dataset would be an excellent resource for future precision medicine applications and functional genomic researches, on the other hands, there are still needs for thoroughly inspection on the tissue expression profiles of multiple alternatively spliced transcripts of human protein-coding genes.

In this report, we have provided a new bioinformatic resource for the study of human protein-coding gene transcript expression profiles in normal human tissues. Protein-coding genes are known to be tissue-specifically modulated with selected transcript isoforms and differentially expressed in certain developmental stages of cell types^19^. We contend that one standard reference transcript per gene may not suffice, especially when considering tissue-specific alternatively spliced isoforms. Researchers should be able to thoroughly investigate the modulations of alternatively spliced transcript isoforms in different tissues. Therefore, we established the TREGT web resource (https://tregt.ibms.sinica.edu.tw/index.php) using normal tissue expression dataset from GTEx. The GTEx project generated useful global RNA expression data within various human tissues of many individuals^20^. In previous studies, different tissue types have been demonstrated to exhibit gene expression signature profiles that are correlated with expression quantitative trait loci (eQTL)^21^. Therefore, we utilized the GTEx data for the expression analysis on alternatively spliced transcript isoforms of human protein-coding genes. Furthermore, using a solo gene expression dataset avoids the complications caused by the mRNA isoform quantitation problems of the NGS transcriptome analysis pipelines when using data from different sources.

## Results

### Human protein-coding transcripts/genes in GTEx dataset

We selected the GTEx dataset because of their expression information obtained from noncancer human tissues. Cancer cells have aberrant splicing isoforms and dysregulated gene expression, which often distort normal mRNA transcript expression information and not suitable for reference transcript collection. The current release of GTEx data contains 199,324 transcripts assigned to 58,219 human genes. There are average 3.42 transcripts per gene. In this study, we did not attempt to discover novel alternatively spliced mRNA isoforms and we relied on the transcript annotations released by GTEx (based on GENCODE version 26). The GENCODE collection has maintained rich and up-to-date information on the alternatively spliced transcripts. To simply define alternatively spliced transcript isoforms, we considered all RNA transcripts (ENSTxxx) with the same Ensembl gene ID (ENSGxxx) as transcript isoforms of the same gene loci. In addition, protein-coding transcripts and genes are defined by the Biotype tag in GENCODE annotation^22^.

However, if only the expressed transcripts are considered, we can further remove 5,178 transcripts with no expression values among all tissues examined. Among the 194,146 expressed transcript, the number of protein-coding gene-derived transcripts is 145,571, which is approximately 75.0% of all expressed transcripts (Table 1). In terms of expressed genes, protein-coding genes (19,591 protein-coding genes) account for approximately 36.6% of the gene pool annotated here. Protein-coding genes generally have higher expression levels and their transcripts are surely observed more in typical RNA-Seq assays^23^. In addition, not all transcript isoforms in protein-coding genes would be translated into protein products. Among the 145,571 transcripts, 80,354 are actual protein-coding transcripts, as defined using the GENCODE biotype tag. Other transcript types in protein-coding genes include the following: processed_transcripts, nonsense_mediated_decay, and retained_intron, etc. These noncoding isoforms in protein-coding genes may possess additional modulation functions^24^. Herein, we mainly investigated the distribution profiles of expressed alternatively spliced isoforms in human protein-coding genes because of their potential biological and functional significance. This bioinformatic resource would be beneficial for exhaustive interrogation of tissue modulated transcript isoform expression within respective protein-coding genes.

**Table 1:** Numbers of transcript and gene in the GTEx dataset. The GTEx V8 dataset was retrieved and processed as described in the Methods.

	GTEx dataset	Expressed all genes	Expressed protein-coding genes
Transcript counts	199,324	194,146 (5.151)^a^	145,571 (6.377)^a^
Gene counts	58,219	53,539 (18.68)^a^	19,591 (47.38)^a^
Transcripts per Gene	3.42	3.63	7.43

^a^Average expression TPM values of expressed transcripts and genes are listed in parentheses.

### Expression of transcript isoforms in protein-coding genes

We were interested in identifying the top-ranked expressed gene transcripts for use as representative standard transcripts. The basic concept was to classify the most abundantly expressed transcript because its expression level would be the greatest in cells, which should indicate that these transcripts are have more possibilities in generating translated protein products. Therefore, the main objective of this study was to identify characteristically expressed transcripts for human protein-coding genes by investigating the expression levels of their spliced transcript isoforms. We ranked the expression level for all transcript isoforms, except the 2756 single transcript protein-coding genes. Therefore, we concentrated our efforts on determining the overall expression pattern for each transcript isoform using the GTEx average expression information of all tissue types.

**Figure 1:**
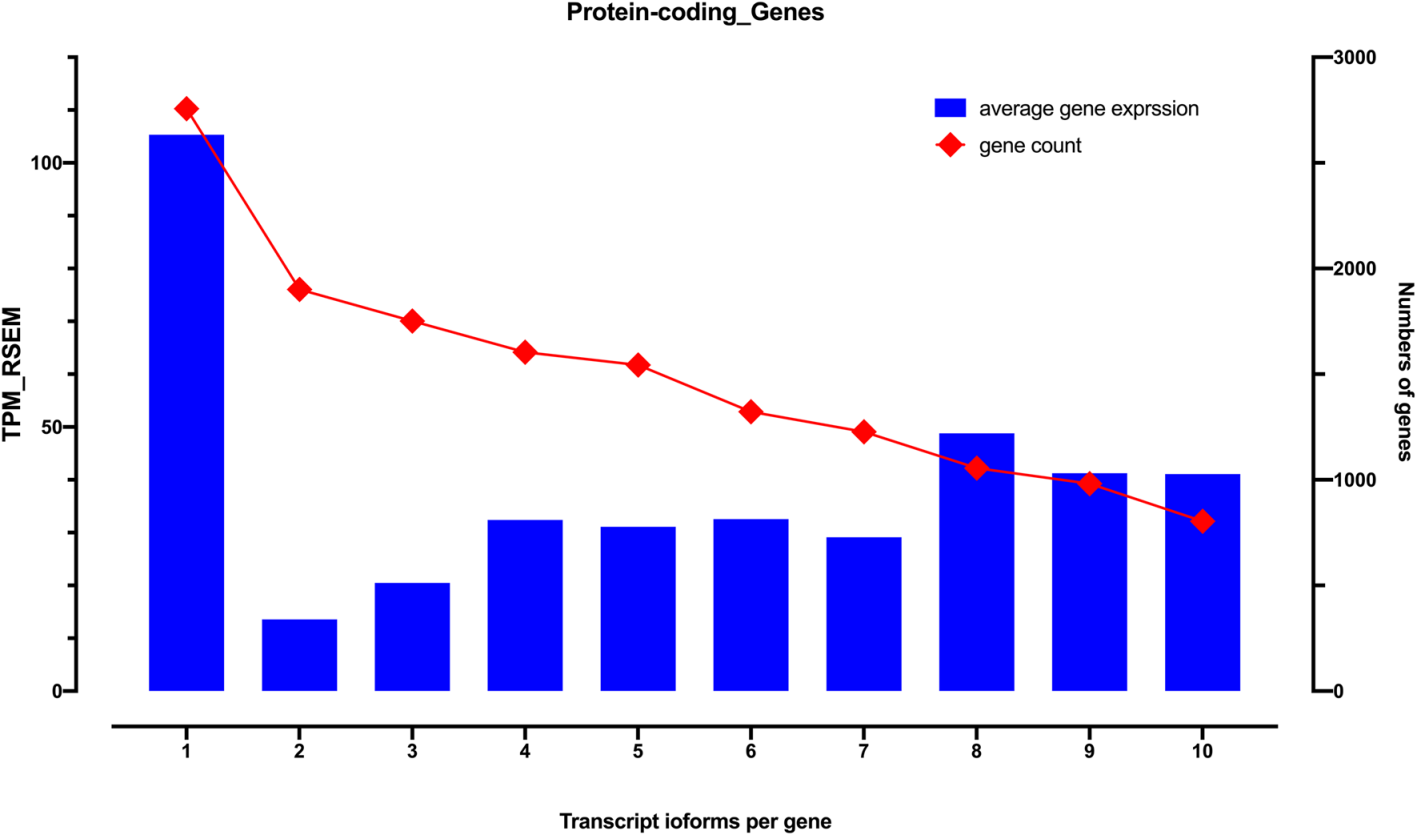
The numbers and average expression levels of protein-coding genes with single transcript gene to 10 transcript isoform genes. There are more single transcript protein-coding genes and they have considerable higher expression levels. Notably, the gene-level expression of protein-coding genes with two and three transcript isoforms was lower than that of other classes.

For all protein-coding genes, the average expression value was 47.38 per gene, whereas the expression value of all expressed genes (protein-coding and noncoding) was reduced to 18.68 (Table 1). The average expression value of transcripts in the protein-coding gene was tabulated as 6.377 per transcript. When counting only protein-coding transcripts in protein-coding genes, their expression level increased to 10.07 per transcript. Instead, the noncoding transcripts in protein-coding genes had an expression level of merely 1.83. Thus, within human protein-coding genes, transcripts with translation potential significantly possess stronger expression capability (10.07 vs 1.83). Because all single transcript genes obviously contain only protein-coding transcripts and single transcript genes have higher expression levels (Fig. 1). Thus, we tried to exclude the single transcript genes in the assessment, the adjusted average expression of protein-coding transcripts (6.684) is still much higher than their noncoding parts in multiple transcript protein-coding genes. We further examined the functional types of protein-coding genes using the DAVID analysis tool. Higher expression levels of the single transcript human protein-coding genes and their enrichment in the olfactory transduction pathway were of particular interest.

### Top-ranked expressed transcripts in protein-coding genes

We ranked the transcript isoforms first by their expression levels and by CDS lengths or transcript lengths if the expression level was identical, using the average expression information of each transcript isoform in protein-coding genes. We then examined the expression characteristics of various ranked transcript isoforms. As illustrated in Table 2, the rank1 transcript had remarkably high expression levels (35.91 average TPM value) compared with rank2 (6.217) and rank3 transcripts (3.072). Again, we further investigated the rank1 transcript isoform expression value by excluding the single transcript genes due to their high expression values. The expression value of rank1 transcript isoforms following adjustment was 24.552, which remained significantly higher than the expression values of other ranked transcripts (p < 0.05). Therefore, top-ranked rank1 transcript could be considered as the predominant transcript isoform in protein-coding genes.

**Table 2:** Average expression TPM values of Rank1 to Rank10 expressed transcripts in protein-coding genes.

Transcript ranks	Protein-coding genes
Rank1	35.910
Rank2	6.217
Rank3	3.072
Rank4	1.872
Rank5	1.292
Rank6	0.973
Rank7	0.762
Rank8	0.621
Rank9	0.520
Rank10	0.446

**Figure 2:**
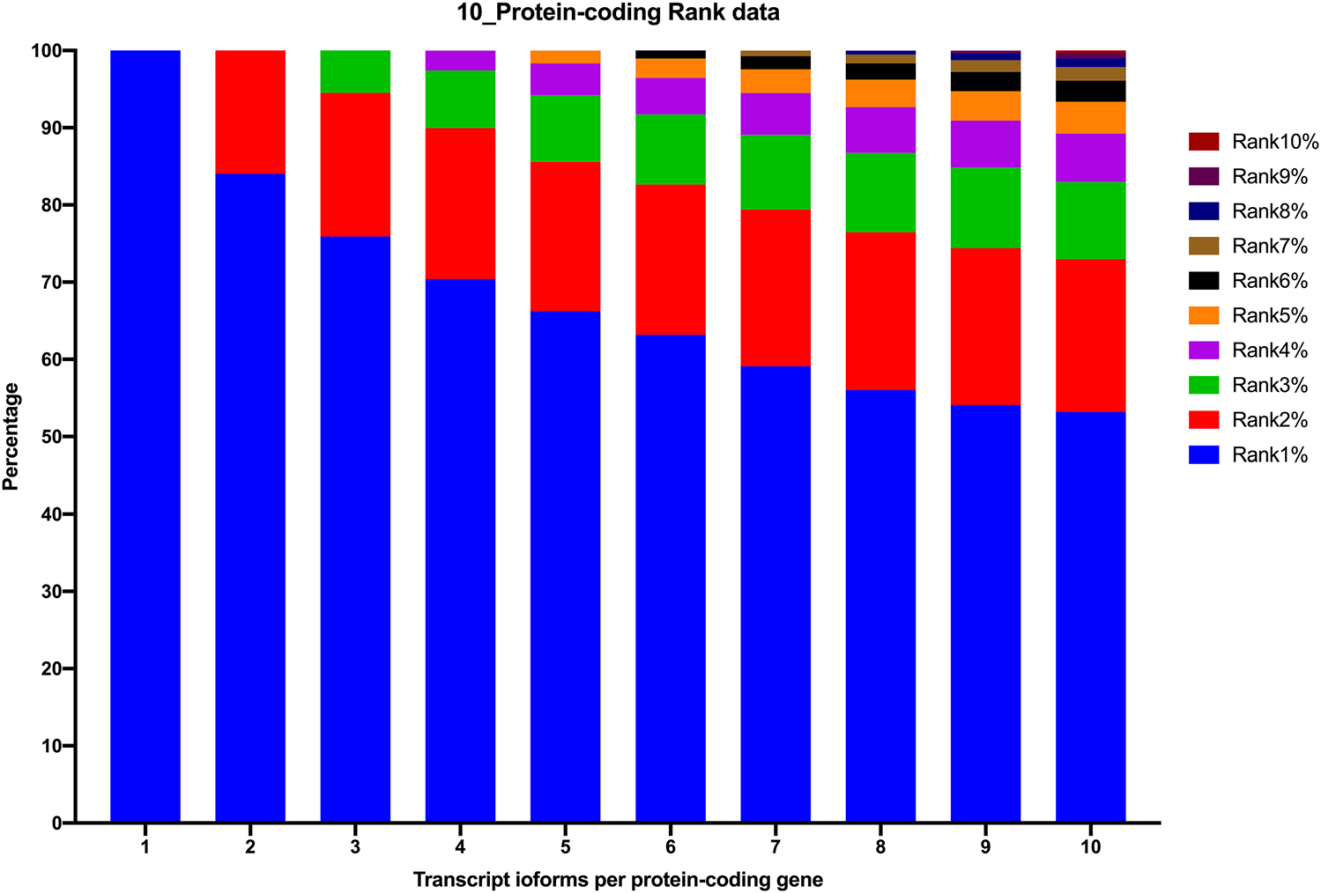
Expression percentages of top-ranked transcript isoforms in human protein-coding genes with 1 to 10 transcripts per gene. We calculated the expression distribution percentages of transcript isoforms in human protein-coding genes. A rank1 transcript isoform was the dominantly expressed transcript isoform, representing over 50% of the expression level in 1 to 10 transcripts per gene. Rank1 to rank5 accounted for over 95% of the gene expression.

We examined the rank1 transcript expression percentage and determined that nearly 70% of the rank1 transcripts were expressed at more than 50% of their respective protein-coding gene expression level. There were more than one quarter of rank1 transcripts whose expression level occupied over 90% of entire gene expression level. Regarding the overall distribution in multiple transcripts, top-ranked transcript isoforms (rank1 to rank5 transcripts) accounted for over 95% of the expression level for protein-coding genes with less than 10 transcript isoforms (Fig. 2). Moreover, the expression coverage of rank1 to rank3 transcripts alone was over 85%. Our data demonstrate that emphasis on the top-ranked expressed transcript collection could be a valuable approach to study representative reference gene transcripts. Intriguingly, approximately 90% of the rank1 transcripts are protein-coding transcripts. Conversely, there are still 2119 rank1 transcripts of protein-coding genes belonging to noncoding biotypes (either processed_transcript or retained_intron). It would be interesting to study more on their biological roles in modulating other protein-coding transcripts.

### Tissue expression patterns of transcript isoforms

**Figure 3:**
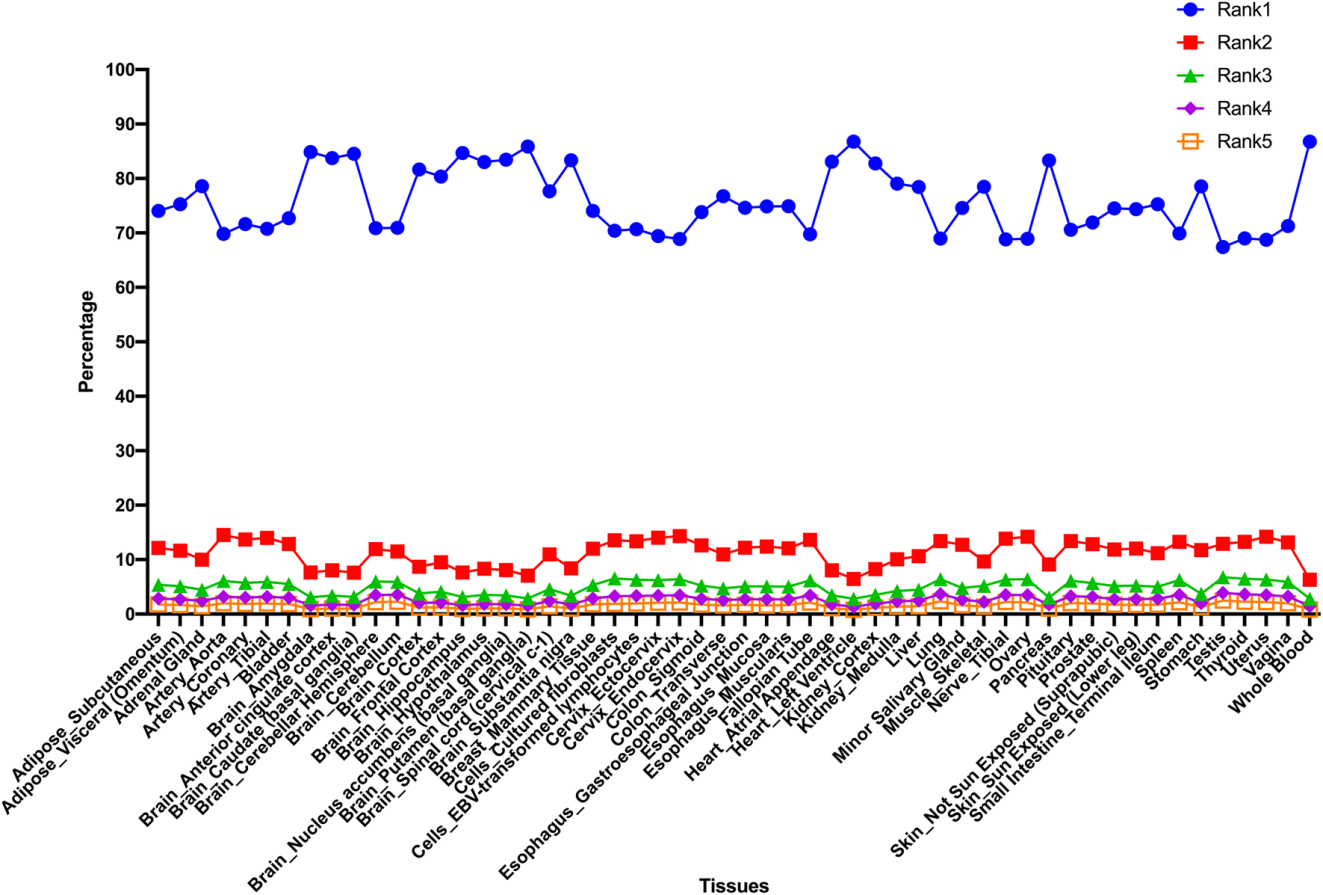
Tissue expression distribution of top-ranked transcript isoforms of human protein-coding genes. Rank1 to rank5 transcript isoform expression percentage in human tissues. We calculated the expression percentage of different ranked transcripts in each gene of various tissue types, and the average expression percentages of these top-ranked transcript isoforms were tabulated by tissue types.

We used the average expression values from all tissue types to rank transcript isoforms despite the GTEx dataset allowing analysis of tissue-specific expression in greater detail. The GTEx dataset contains around 50 tissue types and over 17,000 samples from 948 donors, which provides an unique opportunity to better investigate the tissue patterns of transcript isoforms in protein-coding genes. Numerous protein-coding genes are known to be modulated by developmental stages in a tissue-specific manner. Comprehensively studying the tissue expression patterns among different transcript isoforms is crucial for biologists. Therefore, we further utilized our ranking approach to determine the alternatively spliced transcript isoform expression ranking in each tissue for further interrogations. In Fig. 3, the rank1 top-ranked transcript isoforms were demonstrated to be the dominant transcript type in all tissues. However, rank1 transcript expression exhibited higher expression patterns in particular tissues, such as blood, brain, heart and pancreas tissues, which suggests more diverse gene expression modulation in the highly differentiated cell types of certain tissues (Fig. 3). This is an intriguing and delicate problem. There are multiple considerations in tissue-specific expression modulations, including alternative promoter modulation and regulation, splicing site selection and transcript stability, intron and exon inclusion and exclusion, and even miRNA target sequence modulation^25^. Therefore, protein-coding genes are tissue-specifically modulated with selected transcript isoforms and differentially expressed in certain developmental stages of cell types^26,27^.

**Figure 4:**
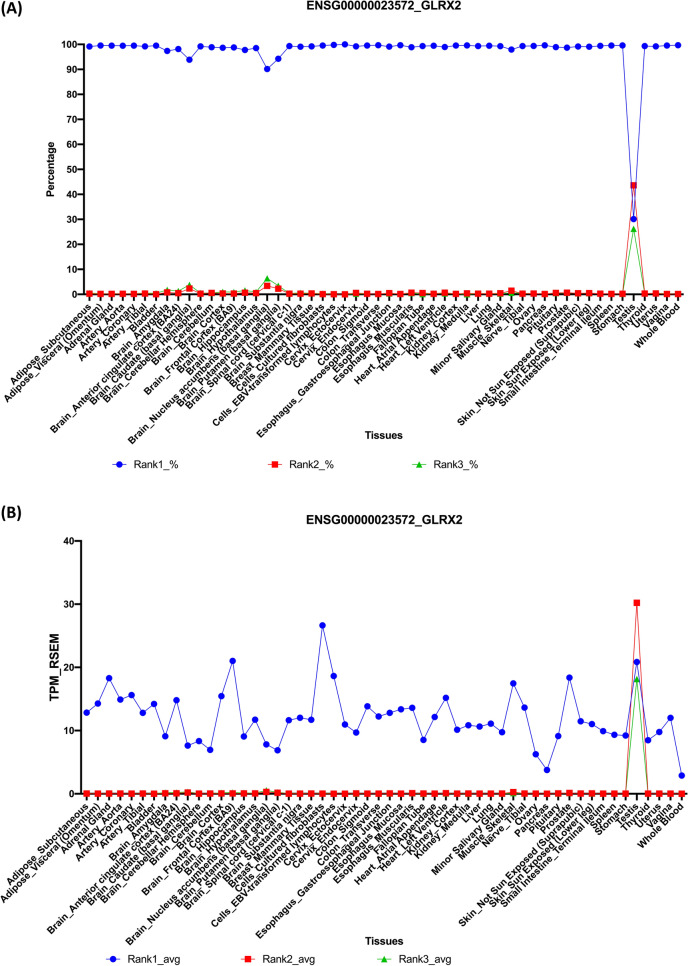
Tissue expression percentage distribution of rank1, rank2, and rank3 transcript isoforms of the human *GLRX2* gene. The *GLRX2* gene is a protein-coding gene for glutaredoxin 2, which has three transcript isoforms. Rank1 is the dominant transcript type in almost all tissues except testis. Rank2 is the top-ranked transcript in testis tissue. **(A)** The expression percentages of rank1 to rank3 transcript isoforms are plotted. **(B)** The expression TPM values of rank1 to rank3 transcript isoforms are plotted.

The present data provided a simplified view of the transcript isoform expression patterns in various tissue types. Modulation on different ranked isoforms indicates a possible functional importance in addition to the expression changes. For instance, in Fig. 4A, the *GLRX2* gene demonstrated a specific alteration in testis tissue expression between different ranked transcript isoforms. The *GLRX2* gene is a protein-coding gene for glutaredoxin 2, which is a thiol-disulfide oxidoreductase that maintains cellular thiol homeostasis and mitochondrial redox homeostasis. The rank1 *GLRX2* transcript isoform (ENST00000367439) expression coverage decreased from almost 100% to approximately 30%, whereas the rank2 (ENST00000472197) and rank3 (ENST00000367440) transcript isoforms increased significantly in testis tissue alone. These differences resulted from the increased expression of rank2 and rank3 transcript isoforms in testis tissue (Fig. 4B). Notably, the rank2 transcript isoform (ENST00000472197) is a processed noncoding transcript, and the rank3 transcript isoform (ENST00000367440) is a protein-coding transcript with different 5′-end coding sequences. Further study into the biological roles of these *GLRX2* transcript isoforms in the testis tissue would be valuable. Previous studies have suggested that the testis-specific alternatively spliced isoforms of the *GLRX2* gene produce different cellular localizations and may have functional implications^28,29^. The *GLRX2* gene case study revealed that a more delicate analysis was needed to investigate the top-ranked transcript isoforms for different respective protein-coding genes. Therefore, new bioinformatic web tools are needed here to further study this type of rank1 transcript switch events.

### Top-ranked transcript isoform display web-interface

**Figure 5:**
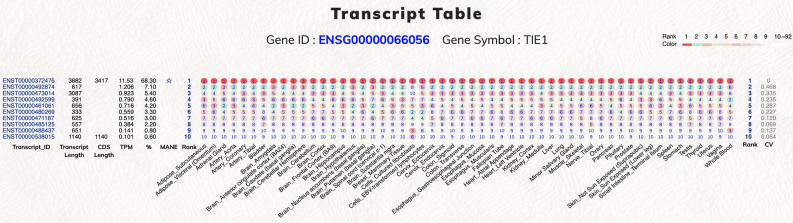
Web user interfaces for top-ranked transcript isoform expression in tissues. The Tie1 protein tyrosine kinase gene has 10 transcript isoforms. Two protein-coding and 8 processed transcripts are illustrated based on their expression ranking. We provided additional information regarding the transcript length, CDS length, TPM value, gene expression percentage, and coefficient of variation value for each transcript isoform. The MANE select transcript is marked by the star symbol. In each tissue, the ranking of each transcript isoform is displayed with color-coded symbols for easy investigation. ENST00000372476 is the rank1 transcript in all tissues.

The GTEx website already provides an excellent web resource for biologists to investigate the expression information at gene and transcript levels and especially eQTL information^30^. The GTEx website also provides an elegant user interface that facilitates investigating the gene expression in all tissues and transcript isoform expression at the exon and junction levels. However, the tissue expression ranking for each transcript isoform is not clearly described. Therefore, we constructed an easy to use web interface for users to examine the top-ranked expressed transcript isoforms in various human normal tissues (https://tregt.ibms.sinica.edu.tw/index.php). One could investigate the top-ranked expressed transcript isoforms here with the convenience of processed alternatively spliced isoforms expression dataset (Fig. 5). In our database, we displayed the transcript expression information by order of overall expression ranking with the specific tissue expression ranking for each transcript isoform. The color-coded ranking icon can assist users to rapidly understand the top-ranked transcript expression distribution. We further calculated the coefficient of variation (CV) with the tissue ranking values for each transcript isoform to provide an additional assessment of tissue expression variation. Therefore, users can easily assess the expression status of each isoform in different tissue types. Besides, we further provided the transcript length, CDS length, TPM value, and gene expression percentage for each transcript isoform. With the simplified information and graphical display interface, users can further investigate the tissue expression status and possible biological implications of each transcript isoform. In addition, top-ranked transcript information (Rank1 to Rank10) can be downloaded separately from our web site for applications in NGS data analysis pipeline. In the downloaded files section, we provide more top-ranked transcript information, including gene ID, transcript ID, gene name, transcript name, transcript count, transcript expression value, gene expression value, transcript/gene expression percentage, GENCODE transcript biotype, transcript length, and CDS length.

Users can query the gene of their interest by the simple query function with office gene symbol. In the advanced query interface, users can further search the genes of their interests by Ensembl gene ID or by NCBI reference RefSeq ID. We also provide an option to interrogate putative rank1 switch expression events, similar to *GLRX2* gene. The putative rank1 switch events were selected using the rank status and rank1 expression coefficient of variation value cutoff. Users can first select the numbers of rank1 tissues tabulated. If there is a rank1 switch event in one particular tissue type, the rank1 count would be ‘53’, as the case of *GLRX2* gene. We provide the choices of ‘53’; ‘52’; ‘51’ and ‘50’ tissue counts. Additional filters can also be implemented: the rank1 expression percentage option can be used to filter the expression rank1 transcripts. There are 944 genes have 53 tissue counts (one rank1 switch event); 540 genes have 52 tissue counts (two rank1 switch events); 333 genes have 51 tissue counts (three rank1 switch events) and finally 296 genes have 50 tissue counts (four rank1 switch events). In the analysis list window, we provide the gene ID, transcript ID, gene name, transcript count; rank1 transcript expression value, rank1 transcript expression percentage. Additional column sorting function can be applied in the analysis list window to assist data viewing. The full gene information can be illustrated by clicking on the Gene ID link. There are additional genes could be observed besides *GLRX2*, such as *SPDYE3* (ENSG00000214300), which is a cell cycle regulator family gene. Interestingly, the rank1 transcript of *SPDYE3* is a noncoding transcript expressed in most of tissues and rank2 protein-coding transcript is elevated only in the testis tissue (Supplementary Fig. 1). For two rank1 switch events, *PITPNB* (ENSG00000180957) gene is found to have the rank2 transcript switch in the cerebellar_hemisphere and cerebellum tissues (Supplementary Fig. 2). *ALDH18A1* (ENSG00000059573) gene is found to have switch events in colon_transverse and small_intestine tissues (Supplementary Fig. 3).

## Discussion

We utilized the GTEx dataset in this study owing to diverse normal tissue types. This would be beneficial in the future NGS data analysis to choose reference transcripts for human protein-coding genes. However, the typical depth of RNA-Seq would be restricted due to large numbers of tissue samples (17,382 samples) in GTEx project. This would confine the interpretation for low-abundance gene transcripts, such as noncoding lncRNA genes. With the increasing knowledge of comprehensive transcriptome composition^31^, it is required to employ dedicated RNA-Seq protocols to enrich mRNA transcripts, such as Capture-Seq or Amplicon-Seq^12,32^. It is suggested that noncoding lncRNA genes could comprise typical alternative splicing mechanisms and more transcript isoforms could be discovered with greater RNA-Seq depth^7^. In our preliminary analysis of GTEx transcripts, the abundance of noncoding genes is much lower (~ 7% coverage) than that of average protein-coding genes (~ 93%). Therefore, the transcriptome coverage of noncoding gene is limited here. It is anticipated that majority of the noncoding genes in GTEx dataset contain fewer alternatively spliced transcript isoforms. Thus, we only concentrate on the protein-coding genes in order to interrogate further on the top-ranked expressed transcripts in tissue samples.

Our findings indicated that top-ranked transcript isoforms are usually the dominant transcripts in protein-coding genes and may have significant biological implications. It was reported previously that one dominant transcript per human protein-coding gene using fewer tissue samples and much less alternatively spliced transcripts analyzed (105,456)^33^. Similar to our findings, it was reported that 85% of the total mRNA expression were contributed from major transcripts, the rank1 transcripts^33^. However, there are still numbers of protein-coding genes that involve more sophisticated transcription modulations between top-ranked and other expressed transcript isoforms. As shown in the *GLRX2* gene (Fig. 4). With increased RNA-Seq depth and tissue types from GTEx, our web tool provides a better understanding on the alternatively spliced transcript isoforms in human protein-coding genes. It is necessary to further study the rank1 transcript switch events in various biological conditions.

There are several regulation possibilities among these spliced transcript isoforms in addition to splice site selections, such as alternative promoters, alternative transcription start sites, and mRNA stability sequences^34–36^. Competitive endogenous RNA (ceRNAs) was reported to be an attractive regulation mechanism and was proposed to link the possible modulation between mRNAs, microRNAs, and lncRNAs^37–39^. The miRNA genes are recognized to be significant gene modulators in development and cancers^40,41^. The alternatively spliced transcript isoforms would likely to have alternative miRNA target sites. The different transcript isoforms may be modulated by groups of tissue-specific miRNAs and produce various tissue expression profiles. Ultimately, balance between different isoform expression levels may affect the final protein product collection inside cells.

Alternatively, spliced transcript isoforms could generate different translated protein products, which would have various biological effects depending on the developmental stage and tissue type. Notably, not all transcript isoforms in protein-coding genes were found to be protein-coding transcripts, and these noncoding transcripts in protein-coding genes may possess additional modulation functions. For example, UV-induced alternatively spliced shorter lncRNA isoform was reported to play functional roles in the *ASCC3* gene under DNA damage pathway^24^. Utilization of the reference transcripts in protein-coding genes and their tissue expression profiles would be helpful in the NGS data analysis pipeline in future transcriptome studies in disease samples.

Therefore, the MANE-select transcript project was established to provide a more unified and representative transcript annotation in protein-coding genes. The highly stringent prerequisites for the MANE-select transcript collection is crucial; however, this approach is time-consuming with manual annotation needed. The recently released MANE v0.9 dataset (released in May 2020) covers 15,569 human protein-coding genes. The GTEx dataset covered 15,121 MANE-select transcripts (GENCODE 26), which were used in this study. Among them, 10,407 MANE-select transcripts are rank1 transcripts, 2,360 MANE-select transcripts are rank2 transcripts, and 963 MANE-select transcripts are rank3 transcripts. Thus, approximately 96% of MANE-select transcripts are covered by the rank1 to rank5 transcripts identified here. This confirmed our findings on the importance of top-ranked expressed transcript isoforms. With our user-friendly web interface, our top-ranked transcript dataset could assist users in further identifying useful transcript isoforms in protein-coding genes for their research projects. Our study provides an alternate web resource for biologists to investigate numerous transcript isoform expression profiles of human protein-coding genes in various human tissues.

## Conclusion

We have provided a comprehensive analysis of expressed transcript isoforms in human protein-coding genes using the GTEx RNA-Seq dataset. We have established a web tool to examine the top-ranked expressed transcript isoforms in various human tissue types with concise transcript information. Users can examine all expressed transcript isoforms and their tissue rankings for any protein-coding genes of interest with easy to use graphical interfaces. In addition, identified rank1 transcript switch genes could have sophisticated transcript modulation in tissue types and possible biological implications.

## Methods

### GTEx data collection

GTEx is a well-established resource database with which biomedical researchers investigate the genetic genotypes and gene expression relationships of different human individuals. The current-release (V8) GTEx datasets were obtained from the GTEx data portal website (https://www.gtexportal.org/home/datasets). The most recent GTEx dataset contains 54 different human tissue types and 17,382 samples from 948 donors. The most abundant tissue (musculoskeletal) has 803 RNA-seq samples, whereas kidney-medulla tissue has only four RNA-seq samples. Sample sizes vary considerably between tissues. Brain tissue has 13 tissue subtypes in the GTEx collection. GENCODE 26 was the annotation standard for this version of the GTEx data release, and the alignment of GTEx RNA-seq was performed using the STAR pipeline. The transcript expression TPM data were used for analysis, and transcript-level quantifications were calculated using RSEM v1.3.0 according to the GTEx web site. We obtained the transcript TPM expression data file (GTEx_Analysis_2017-06-05_v8_RSEMv1.3.0_transcript_tpm.gct.gz), and the total number of transcripts was 199,324, which were assigned to 58,219 human genes. We initiated the study by first remove 5,178 non-expressed transcripts from the 199,324 transcripts. In this study, we defined all expressed transcripts under the same Ensembl gene ID as alternatively spliced transcript isoforms. We further defined protein-coding transcripts and genes by the Biotype feature. Therefore, there are 145,571 transcripts assigned to 19,591 protein-coding genes, and the remaining 48,575 transcripts were assigned to 33,948 noncoding genes.

### Expression ranking of transcript isoforms

The GTEx transcript TPM expression data file was initially processed using Python scripts, and the transcript expression data were stored separately based on different tissue types. We classified the transcript types according to the biotype labels. Protein-coding genes were defined by their protein-coding transcripts comprised. The top-ranked expressed transcript isoform was investigated to increase understanding of the transcript isoforms and their functional implications. The average expression information of each transcript isoform in protein-coding genes was used to rank the transcript isoforms by expression level, and CDS lengths or transcript lengths were used if the expression levels were identical. The expression characteristics of various ranked transcript isoforms were then examined. GraphPad Prism software (version 8.4) was used for part of the data analysis and graph data illustration (https://www.graphpad.com). Python and R packages (wilcox.test; t.test) were used for all statistical analyses, including data preprocessing and descriptive analysis (partial python scripts were listed in the supplementary file). Inferential analyses were also performed, including the Mann–Whitney U test, to examine the differences in transcript isoform expression between protein-coding genes as described previously^42^. Statistical significance was set at 0.05. The variance between the transcript ranking among 54 different tissues was calculated based on the coefficient of variation (CV value) of all transcript isoforms in each tissue to analyze whether transcript isoforms in protein-coding genes exhibited tissue-specific expression profiles. We then identified putative significant Rank1 switch event genes by the rank1 TPM CV value cutoff as well as the numbers of rank1 tissues in all protein-coding genes (50 to 53 tissue counts). *GLRX2*, *SPDYE3*, *PITPNB* and *ALDH18A1* genes were randomly selected as examples.

### NCBI RefSeq and MANE-select transcripts

The NCBI RefSeq gene and transcript ID were retrieved and extracted from the gene2ensembl file downloaded from NCBI web site. We utilized the Ensembl gene ID and RefSeq transcript ID (NM_xxxxxx) as the reference key ID. The MANE.GRCh38.v0.9.summary.txt file was obtained from the NCBI website. The MANE-select transcript information was extracted and compared with the GTEx dataset. We utilized the Ensembl gene ID and transcript ID as the comparison reference. There are 15,529 MANE-select gene and transcript records, and matched gene ID and transcript ID between MANE-select and GTEx v8/GENCODE 26 dataset were retried for analysis. In total, 15,529 genes with matched gene ID were found in both datasets. We further compared the MANE-select transcripts in the matched gene records.

### TREGT website construction

The web database server presented in this study is running in the Docker environment and operates on CentOS 7.x Linux. It was implemented on an Apache webserver with PHP in conjunction with the MySQL database for data as described previously^43^. Access to our web database is free with no restriction or login account required. All data on the webserver are stored in a flat file format and loaded in our MySQL database for visualization. The D3 javascript library is used for summary data presentation and to enhance the interactive user-friendly interface^43^. All transcript isoform ranking information is publicly available at https://tregt.ibms.sinica.edu.tw/index.php . In addition, the top-ranked expressed transcripts (Rank1 to Rank10) are available for download from the website.

## Supplementary information

Supplementary Information.
