# Supplementary material for: Top-ranked expressed gene transcripts of human protein-coding genes investigated with GTEx dataset
Source: Sci Rep. 2020 Oct 1;10:16245. doi: 10.1038/s41598-020-73081-5 (PMC7530651; doi:10.1038/s41598-020-73081-5)
Supplement: Supplementary file 1 — Supplementary Information. [file 41598_2020_73081_MOESM1_ESM.pdf]

Supplementary Information

Top-ranked expressed gene transcripts of human protein-coding genes investigated with GTEx dataset

Kuo-Feng Tung[1], Chao-Yu Pan[1, 2], Chao-Hsin Chen[1] and Wen-chang Lin[1, 2, *]

[1]Institute of Biomedical Sciences, Academia Sinica, Taipei, Taiwan, R.O.C.

[2]Institute of Biomedical Informatics, National Yang-Ming University, Taipei, Taiwan, R.O.C.

Python scripts:

1. reading GTEx Tissue data:

```
import pandas as pd

GTEx_Tissue = pd.read_csv('/home/GTExv8/GTEx_Analysis_2017-06-05_v8_RSEMv1.3.0_transcript_tpm.csv', delimiter= '\t', low_memory=False)
```

2. Delete rows with all zero values:

```
GTEx_subTissue['sum'] = GTEx_Tissue.iloc[0:,2:].astype(float).sum(axis=1)
GTEx_subTissue_nonZero = GTEx_subTissue[GTEx_subTissue['sum'] != 0]
GTEx_subTissue_nonZero.to_csv('/SNP_data/GTExv8/GTEx_Analysis_2017-06-05_v8_RSEMv1.3.0_transcript_tpm_nonZero_194146.csv')
```

3. Selecting protein-coding genes:

```
import os
import pandas as pd

GTEx_subTissue_nonZero = pd.read_csv('/data/GTExv8/54_SubTissue_194146/GTEx_Analysis_2017-06-05_v8_RSEMv1.3.0_transcript_tpm_nonZero_194146.csv', delimiter= ',', header=None, low_memory=False)

path = '/data/Gencode26/'
gencode_v26_protein_coding_genes = pd.read_csv(path + 'gencode.v26.protein-coding_genes.csv')
gencode_v26_protein_coding_genes_list = list(gencode_v26_protein_coding_genes['protein-coding gene_id'])
gencode_v26_protein_coding_genes_list[0]= 'tissue_gene'
gencode_v26_protein_coding_genes_list[1]= 'sub_tissue_gene'
gencode_v26_protein_coding_genes_list[2]= 'gene_id'
GTEx_54_subTissue_protein_coding =
GTEx_subTissue[GTEx_subTissue['tissue_gene'].isin(gencode_v26_protein_coding_genes_list)]
GTEx_54_subTissue_protein_coding.to_csv('54_SubTissue/GTEx_54_subTissue_protein_coding.csv')
```

4. Create 54 sub-tissues data:

```
GTEx_54_subTissue_protein_coding_sample = GTEx_54_subTissue_protein_coding.loc[2:, :]

SubTissue_list = [x for x in GTEx_54_subTissue_protein_coding.columns if "." not in x]
del SubTissue_list[0:3]
del SubTissue_list[-1]
```

```
Unique_SubTissue_list = list(set(SubTissue_list))

for tissue in Unique_SubTissue_list:
    Tissue = GTEx_54_subTissue_protein_coding_sample.filter(like=tissue)
    Tissue.insert(0, "tissue", GTEx_54_subTissue_protein_coding['tissue'], True)
    Tissue.insert(1, "tissue_gene", GTEx_54_subTissue_protein_coding['tissue_gene'], True)
    tissue_name = tissue.replace(' - ', '_')

    if not os.path.exists('54_SubTissue/GTEx_54_SubTissue_protein_coding_split_54SubTissues'):
        os.makedirs('54_SubTissue/GTEx_54_SubTissue_protein_coding_split_54SubTissues')
        print ('create 54_SubTissue/GTEx_54_SubTissue_protein_coding_split_54SubTissues folder')


Tissue.to_csv('54_SubTissue/GTEx_54_SubTissue_protein_coding_split_54SubTissues/GTEx_SubTissue_' +
tissue_name + '.csv', header=False, index=False, )
```

5. Calculating 54 sub-tissues sum, mean and group ranking :

```
import os
import pandas as pd

tissue_dir = 'GTEx_54SubTissue'

fname = [name for name in os.listdir(tissue_dir) if '.csv' in name]

for file in fname:
    Sub_Tissue = pd.read_csv('GTEx_54SubTissue/' + file)
    SubTissue_data = Sub_Tissue
    SubTissue_data.columns = SubTissue_data.iloc[0]
    SubTissue_data.drop(SubTissue_data.index[:1], inplace=True)

    SubTissue_data['sum'] = SubTissue_data.iloc[0:,2:].astype(float).sum(axis=1)
    SubTissue_data['mean'] = SubTissue_data.iloc[0:,2:-1].astype(float).mean(axis=1)

    SubTissue_data["group_rank"] =
SubTissue_data.groupby("gene_id")["sum"].rank(ascending=0,method='dense')

    SubTissue_data = Sub_Tissue.groupby(['gene_id', 'transcript_id'])
```

```
SubTissue_data.first().to_csv('GTEx_54SubTissue_groupby/' + file.replace('.csv', '') + '_groupby.csv' )
state_office = Sub_Tissue.groupby(['gene_id', 'transcript_id', 'sum', 'mean', 'group_rank']).agg({'mean':
'sum'})

state_pcts = state_office.groupby(level='gene_id').apply(lambda x: 100 * x / float(x.sum()))
state_pcts.rename(columns={'mean':'percentage'}, inplace=True)
state_pcts.fillna(0, inplace=True)

state_pcts.to_csv('GTEx_54SubTissue_ranking/' + file.replace('.csv', '') + '_percentage.csv' )
```

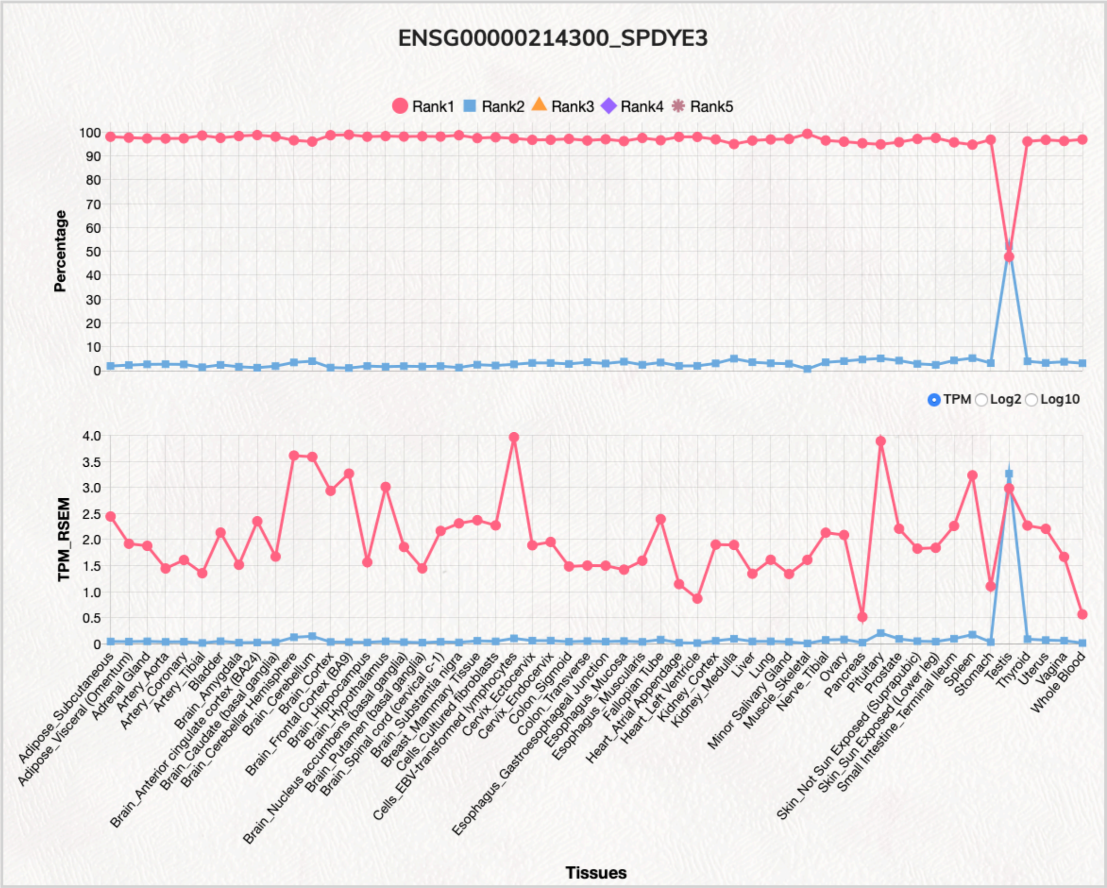

Supplementary Figure 1. Tissue expression percentage distribution of rank1 to rank5 transcript isoforms of the human *PITPNB* gene. The *PITPNB* gene is a protein-coding gene for phosphatidylinositol transfer protein beta, which has eleven transcript isoforms. Rank1 transcript is the dominant transcript type in most tissues. Rank2 transcript has elevated expression in brain tissues and is the top-ranked transcript in brain cerebellar hemisphere and brain cerebellum tissues. The expression percentages of rank1 to rank5 transcript isoforms are plotted in the upper panel. The expression TPM values of rank1 to rank5 transcript isoforms are plotted in the lower panel.

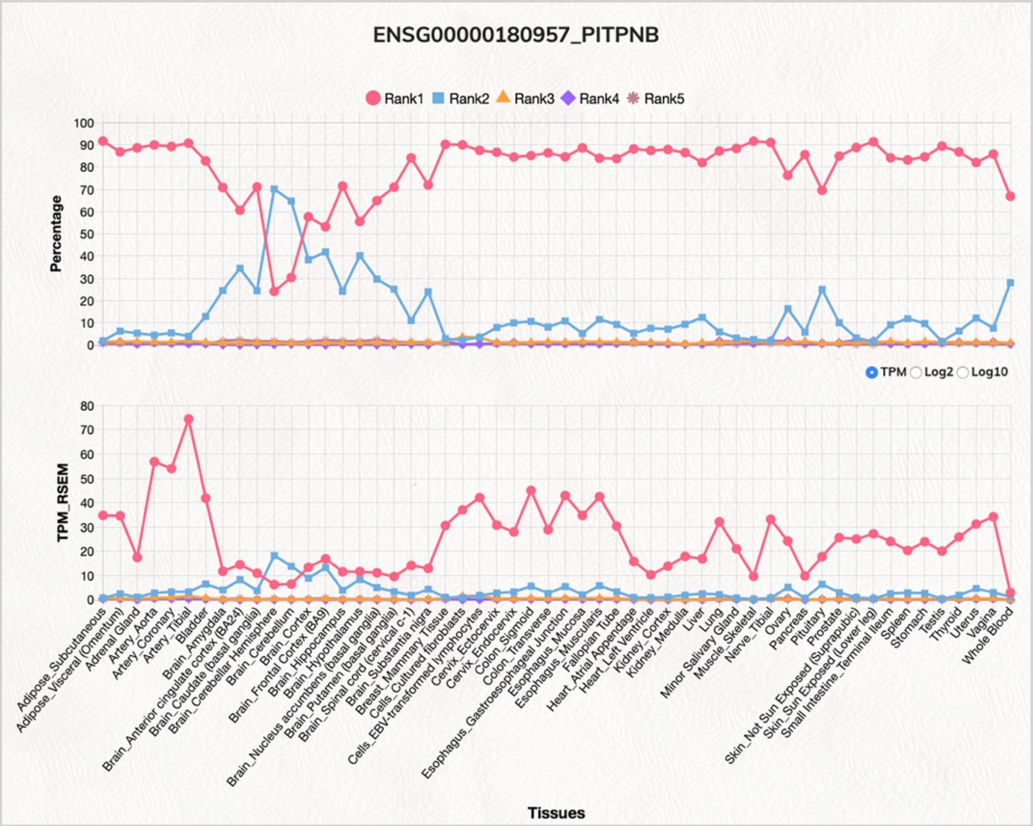

Supplementary Figure 2. Tissue expression percentage distribution of rank1 to rank5 transcript isoforms of the human *PITPNB* gene. The *PITPNB* gene is a protein-coding gene for phosphatidylinositol transfer protein beta, which has eleven transcript isoforms. Rank1 transcript is the dominant transcript type in most tissues. Rank2 transcript has elevated expression in brain tissues and is the top-ranked transcript in brain cerebellar hemisphere and brain cerebellum tissues. The expression percentages of rank1 to rank5 transcript isoforms are plotted in the upper panel. The expression TPM values of rank1 to rank5 transcript isoforms are plotted in the lower panel.

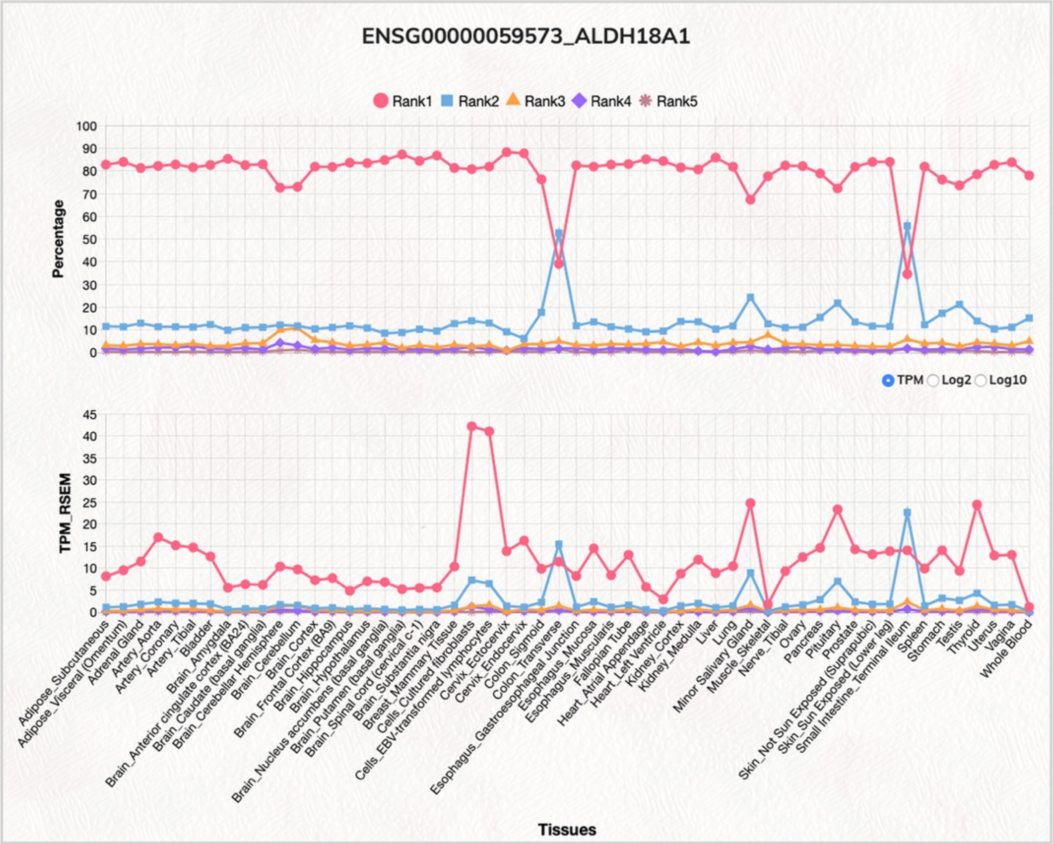

Supplementary Figure 3. Tissue expression percentage distribution of rank1 to rank5 transcript isoforms of the human *ALDH18A1* gene. The *ALDH18A1* gene is a protein-coding gene for aldehyde dehydrogenase 18 family member A1, which has five transcript isoforms. Rank1 transcript is the dominant transcript type in most tissues. Rank2 transcript has elevated expression in colon and small intestine tissues. The expression percentages of rank1 to rank5 transcript isoforms are plotted in the upper panel. The expression TPM values of rank1 to rank5 transcript isoforms are plotted in the lower panel.
